# Comprehensive analysis of alternative polyadenylation regulators concerning CD276 and immune infiltration in bladder cancer

**DOI:** 10.1186/s12885-022-10103-7

**Published:** 2022-09-29

**Authors:** Ming Xiong, Wencheng Li, Longwang Wang, Liang Chen, Zhaohui Chen, Chengcheng Wei, Futian Zhang, Jiawei Chen, Gallina Kazobinka, Jun Zhao, Teng Hou

**Affiliations:** 1 Department of Urology, Union Hospital, Tongji Medical College, Huazhong University of Science and Technology, Wuhan, China; 2 Department of Urology, The First Affiliated Hospital of Nanchang University, Nanchang, 330006 China

**Keywords:** Alternative polyadenylation, Bladder cancer, Immune infiltration, Immunotherapy target, CPSF3, CD276

## Abstract

Alternative polyadenylation (APA) is emerging as a crucial regulatory mechanism in bladder cancer (BC), while it remains elusive whether APA influences the tumor immune microenvironment (TIME) in BC. We identified two distinct subtypes of BC by APA-related regulatory genes expression profiles. The two subtypes have different pathological grades, prognostic outcomes, tumor immune infiltration characteristics, and pathway enrichment. Subsequently, CPSF3 was identified as a potential immune infiltration-related gene in BC. Highly expressed CPSF3 was positively correlated with unfavorable prognosis and high CD276 expression in BC. Moreover, we verified the expression of CPSF3 in BC tissues and cell lines by qRT-PCR. In conclusion, the study indicates that APA regulatory factors play an important role in immune infiltration of BC, and that CPSF3 was a potentially prognostic marker and immunotherapy target for BC.

**Supplementary Information:**

The online version contains supplementary material available at 10.1186/s12885-022-10103-7.

## Introduction

Bladder cancer (BC) is the second common genitourinary malignancy [1]. Approximately 573,278 new cases and 212,536 deaths occurred each year worldwide (Sung, Ferlay, et al. 2021). BC can be categorized as non–muscle-invasive bladder cancer (NMIBC) and muscle-invasive bladder cancer (MIBC) [2]. Although 75% of patients present with NMIBC at first diagnosis, 20% of patients will eventually progress to muscle-invasive or metastatic disease [3]. In recent years, immune checkpoint inhibitors (ICIs) have shown unprecedented benefits in BC patients, and the use of ICIs has become first-line treatment for metastatic BC. However, patients with metastatic BC do not obtain long-lasting clinical benefit benefits from immunotherapy [4]. Therefore, an in-depth understanding of the regulatory mechanism of tumor immune microenvironment (TIME) and immunotherapy in BC may help improve treatment efficacy.

Alternative polyadenylation (APA) is an important posttranscriptional regulatory mechanism that recognizes different polyadenylation signals on transcripts, resulting in transcripts with distinct 3'-untranslated regions (3'UTRs). At least 70% of human genes have more than two transcript isoforms with alternative 3'UTRs [5, 6]. It has been shown that APA plays role in key biological processes like gene regulation, cell proliferation, senescence, and also in various human diseases [7]. Recently, APA has been demonstrated to be associated with cancer development [8]. For example, Fischl et al. identified hnRNPC as a critical role in the establishment of APA profiles characteristic for colon cancer progression [9]. Venkat et al. found APA drives oncogenic gene expression in pancreatic ductal adenocarcinoma [10]. Our previous study demonstrated that NUDT21 inhibited BC growth and metastasis through APA [11]. While there are accumulating research involving intrinsic mechanisms of APA in multiple tumors, no investigators have studied the role of APA regulators in the TIME of BC.

In this study, we analyzed the expression profiles of 26 APA regulatory factors and their roles in BC. In addition, we identified two distinct subtypes of BC by consensus clustering analysis, and compared the inter-tumor heterogeneity and diversity in TIME’s composition between the two subtypes. Moreover, we identified CPSF3 as a potential key regulatory gene in immune infiltration in BC. High CPSF3 expression was related to an unfavourable prognosis in bladder cancer. In summary, our study provides new insight into the potential role of APA in TIME and immunotherapeutic approaches to bladder cancer treatment.

## Results

### Expression of APA regulatory factors in BC

**Fig. 1:**
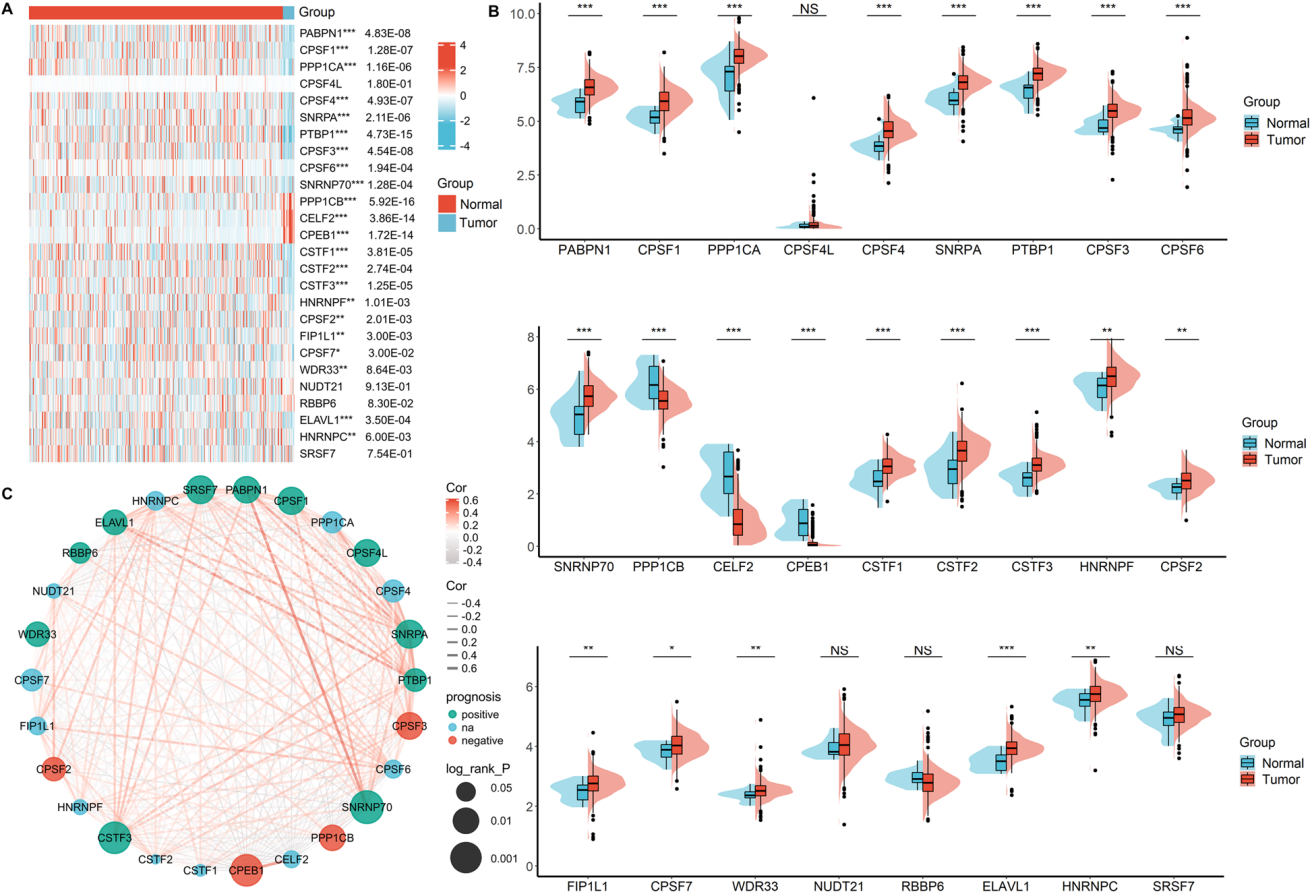
The expression distribution, correlation, and prognostic values of APA regulators in bladder cancer (BC) patients. Heat map (**A**) and violin plots (**B**) of alternative polyadenylation regulators in BC compared to normal tissues. *P*-values are marked after gene symbol in heat map. (**C**) Spearman correlation and prognostic values of APA regulators in BC.The red and grey line respectively represents the positive and negative correlation. The red and green dot represents bad and good prognosis, respectively. The larger the circle, the smaller the prognosis log rankp. *p < 0.05, **p < 0.01, and ***p < 0.001

A total of 26 APA regulatory factors derived from previous study were defined as key APA regulator genes because of their pivotal roles in regulating APA [12, 13]. To understand the biological roles of APA regulatory factors in BC, we analysed the expression profiles of 26 APA regulatory factors in 408 BC patients and 19 normal control from the TCGA dataset. Different expressed genes were found in BC and non-tumorous tissues, including significantly upregulated genes PABPN1, CPSF1, PPP1CA, CPSF4, SNRPA, PTBP1, CPSF3, CPSF6, SNRNP70, CSTF1, CSTF2, CSTF3, HNRNPF, CPSF2, FIP1L1, CPSF7, WDR33, ELAVL1, HNRNPC, and significantly downregulated genes PPP1CB, CELF2, and CPEB1. No significant difference was observed regarding the expression of CPSF4L, NUDT21, RBBP6, SRSF7 (Figs. 1A,B). Moreover, the correlation analysis showed that some APA regulatory genes expression was tightly correlated and prognostically beneficial in BC (Figs. 1C). These results demostrated that APA regulatory factors might be involved in the development and progression of BC.

### Consensus clustering analysis of APA regulatory factors

**Fig. 2:**
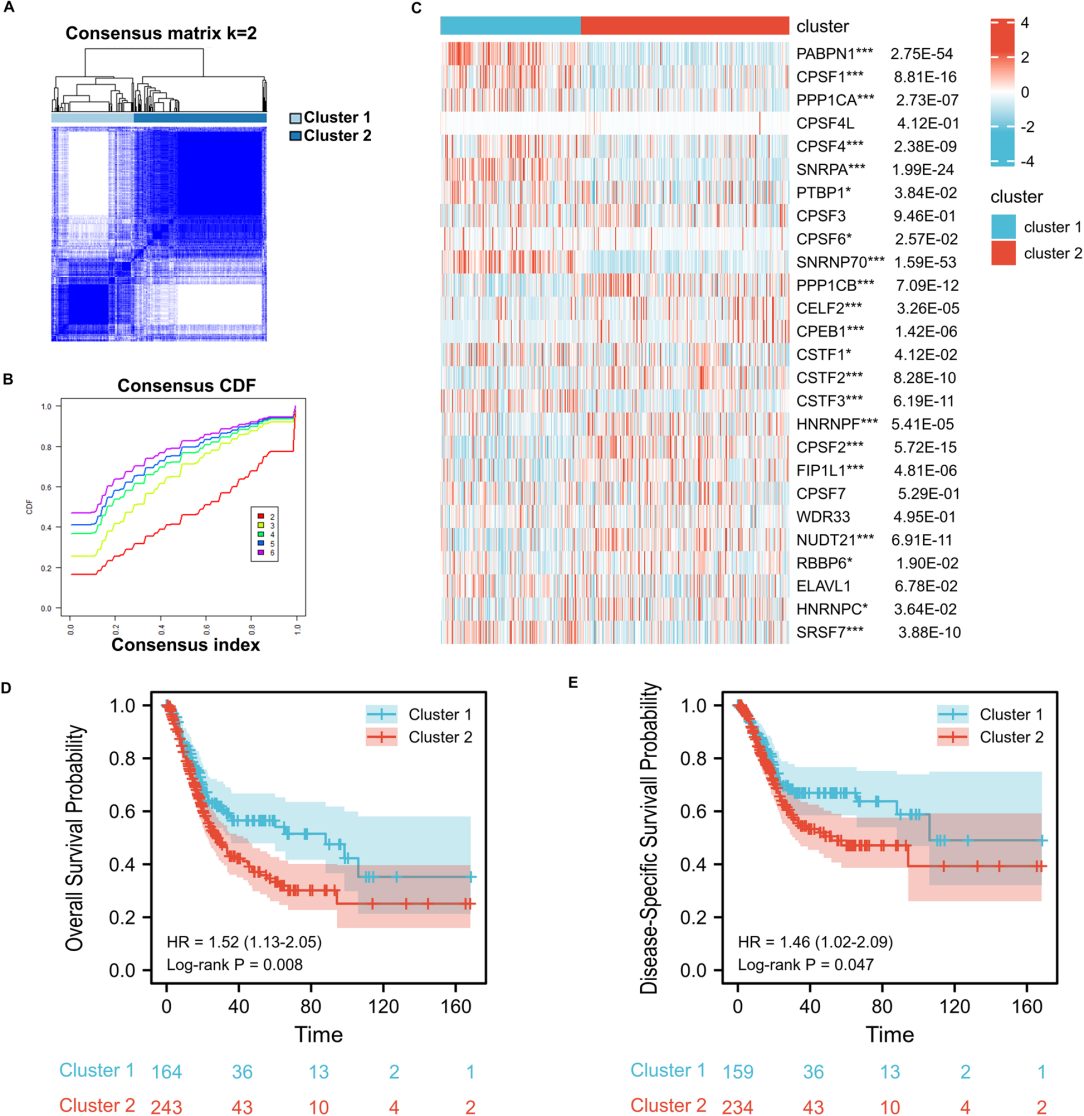
Differential expression pattern of APA regulators and survival in two BC subtypes. **A** Consensus clustering matrix for k = 2. **B** Cumulative distribution function curves fork = 2–6. (**C**) Heat map visualized the expression patterns of f APA regulators in two BC subtypes or clusters. **D**, **E** The Kaplan–Meier curves show the overall survival (**D**) and the disease-specific survival (**E**) for two clusters of BC patients. *p < 0.05, **p < 0.01, and ***p < 0.001

To characterize the molecular profile of BC, consensus clustering analysis was applied based on the gene expression profiles and the proportion of ambiguous clustering measures. K-means clustering (K = 2 clusters) was adopted as a suitable parameter from 2 to 6 (Figs. 2A and B; Supplementary Fig. 1A–E). According to cluster analysis, 408 TCGA BC patients were segregated into cluster1 (n = 164) and cluster 2 (*n* = 244). Differential expression analyses of APA regulatory factors were performed between the 2 subtypes. Compared with cluster2, 10 of 26 factors showed statistically high expression, while 11 factors were in low expression levels in cluster 1. Of the remaining 5 factors, there was no significant difference between the two clusters (Fig. 2C). Then the clinical characteristics from both clusters were grouped for differentiation analysis (Table 1). Cluster 1 displayed lower Asian percentage (*P* < 0.001) and grade (*P* < 0.01). Significant differences were not found in age, gender, and pathologic stage. Furthermore, survival curve showing overall survival (OS) and disease-specific survival (DSS) were significant for cluster 1 compared with cluster 2 (OS Log-rank *P* < 0.01, DSS Log-rank *P* < 0.05) (Fig. 2D and E). Principal component analysis (PCA) was used to further verify the expression difference between the two subtypes (Supplementary Fig. 1F). These results indicated that the expression profiles of clustered subtypes are significant differences.

**Table 1:** Clinical characteristics of two clusters of BC patients

	Feature	Cluster 2	Cluster 1	*P*-value
Age	Meidan (IQR)	69.4 (60.9, 76.5)	69 (60.7, 76.9)	0.986
Gender, n (%)	Female	60 (14.7%)	47 (11.5%)	
	Male	184 (45.1%)	117 (28.7%)	0.423
Race, n (%)	Asian	14 (3.6%)	30 (7.7%)	
	Black or African American	13 (3.3%)	10 (2.6%)	
	White	207 (52.9%)	117 (29.9%)	< 0.001
pathologic stage, n (%)	Stage I	2 (0.5%)	0 (0%)	
	Stage II	69 (17%)	61 (15%)	
	Stage III	82 (20.2%)	58 (14.3%)	
	Stage IV	90 (22.2%)	44 (10.8%)	0.066
Grade, n (%)	High Grade	236 (58.3%)	148 (36.5%)	
	Low Grade	6 (1.5%)	15 (3.7%)	0.006

### Association of APA regulatory factors with CD276 expression and immune cell infiltration in BC

**Fig. 3:**
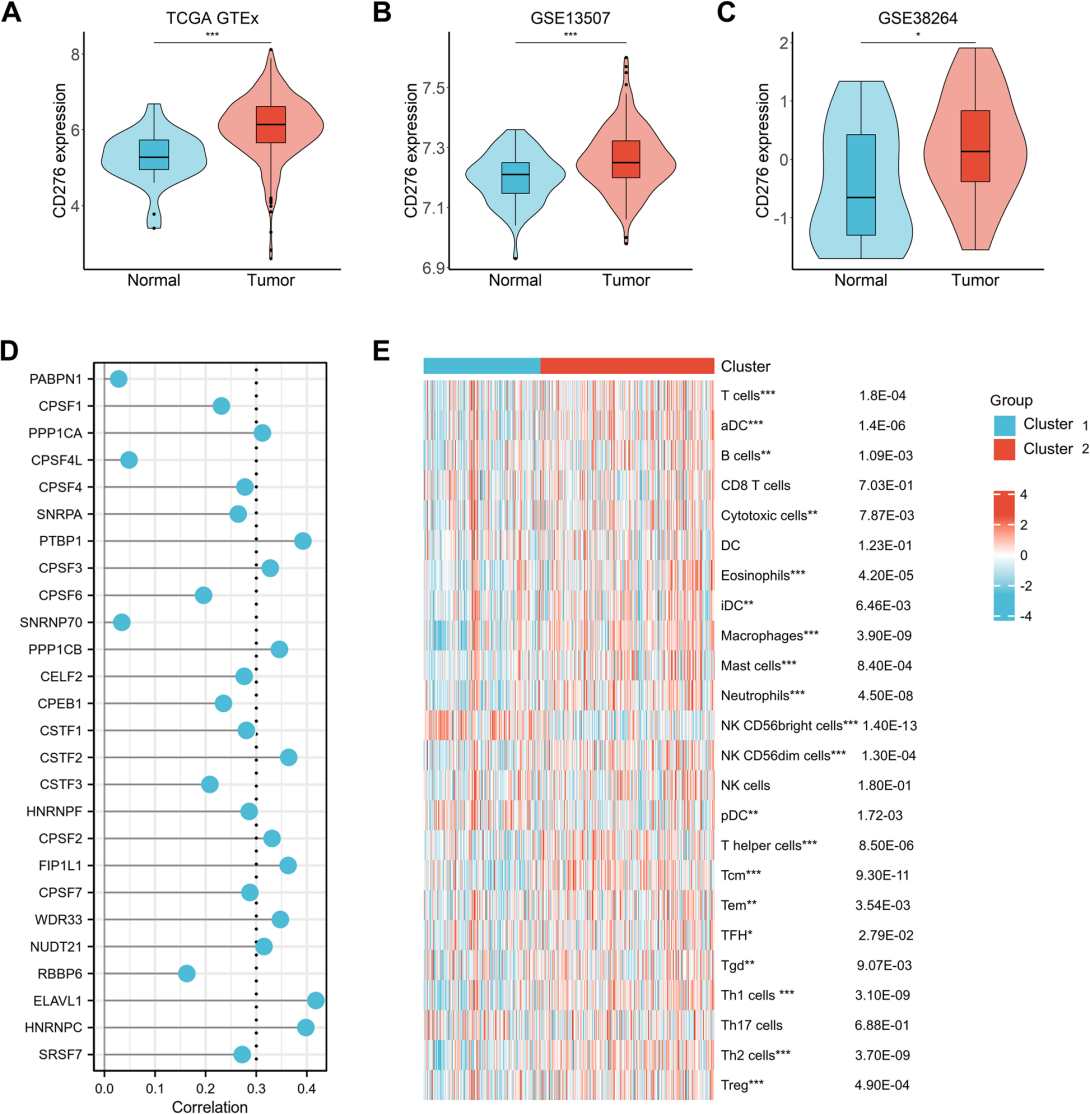
Association of CD276 with APA regulators and the differential immune infiltration in two BC subtypes. **A**, **B** The expression level of CD276 in TCGA-BLCA (**A**) and GSE13507 (**B**), and GSE38264 (**C**). **D** The correlation of CD276 with APA regulators in the TCGA-BLCA cohort. **E** The infiltrating levels of various immune cell types in two subtypes in the TCGA-BLCA cohort. *p < 0.05, **p < 0.01, ***p < 0.001

**Fig. 4:**
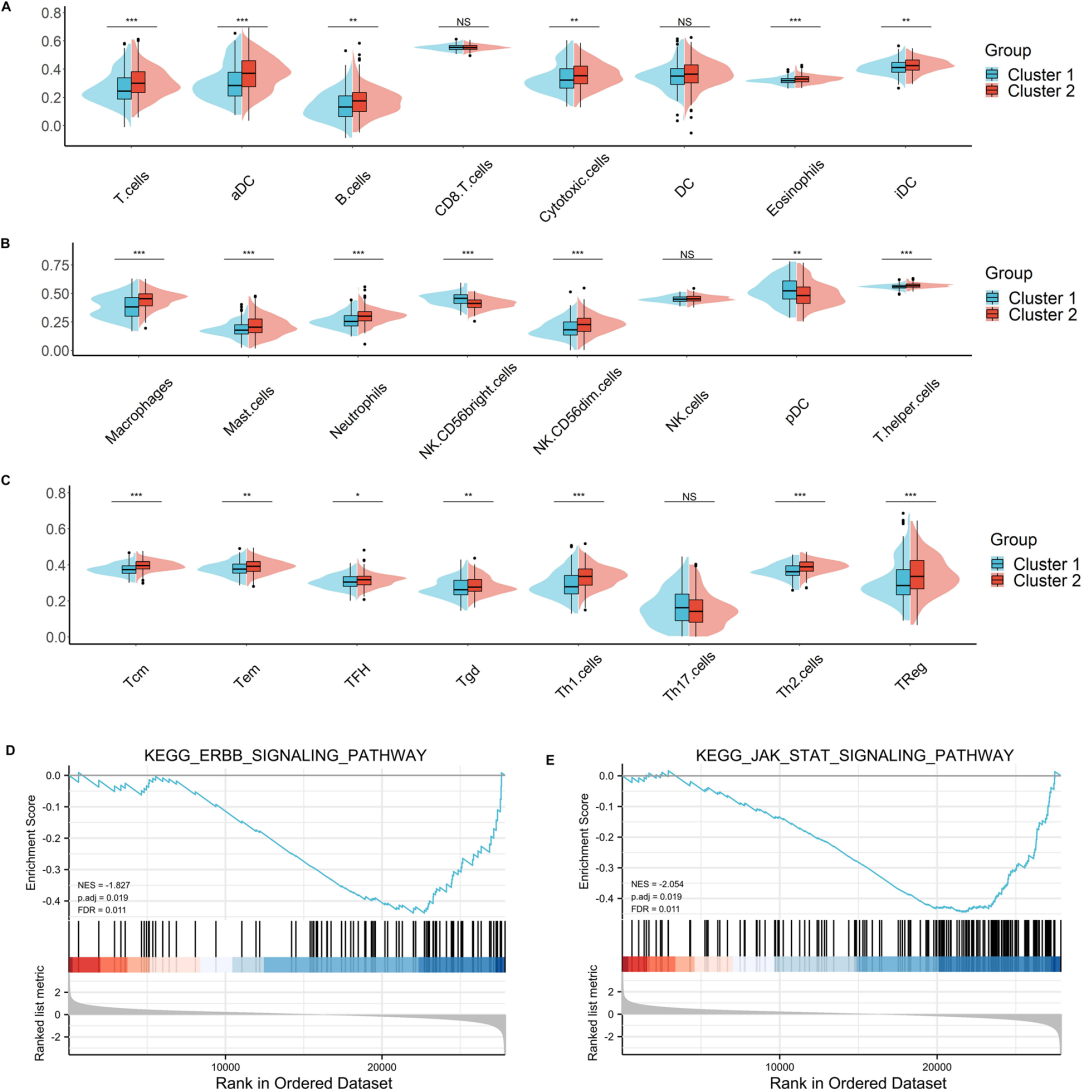
The differences of immune cell infiltration levels in two bladder cancer (BC) subtypes. **A**–**C** The infiltration level of the T cells, aDC, B cells, Cytotoxic cells, Eosinophils, iDC, Macropahges, Mast cells, Neutrophils,NK CD56dim cells, T helper cells, Tcm, Tem, TFH, Tgd, Th1 cells, Th2 cells, and TReg were downregulated in cluster 1 BC patients. The infiltration levels of the NK CD56^bright^ cells, pDC were upregulated in cluster 1 BC patients. **D**, **E** Gene set enrichment analysis indicated that ERBB signaling and the JAK-STAT signaling pathways are significantly enriched in cluster 1. NES, normalized enrichment score;p.adj; FDR, false discovery rate. *p < 0.05, **p < 0.01, ***p < 0.001

CD276 was a novel immune checkpoint molecular implicated in tumor immune escape. To examine the expression of CD276 in BC, data from the TCGA-BLCA and two GEO cohorts was analyzed (Figs. 3A-C). Compared with normal tissues, BC tissues showed higher expression level of CD276 (*P* < 0.001, *P* < 0.001 and *P* < 0.05, respectively). Then we investigated the correlation of APA regulatory factors with CD276 (Figs. 3D). The results showed that CD276 was positively correlated with 11 factors (PPP1CA, PTBP1, CPSF3, PPP1CB, CSTF2, CPSF2, FIP1L1, WDR33, NUDT21, ELAVL1, HNRNPC, correlation coefficient > 0.3 and *P* < 0.05) and negatively correlated with 0 factors. To further explore the roles of APA regulatory factors in BC immune microenvironment, tumor-infiltrating immune cell densities were calculated based on the two clustered subtypes (Figs. 3E). The majority of immune cell types exhibited higher percentages in cluster 2 compared with cluster 1. In particular, cluster 1 showed higher proportions of NK CD56^bright^ cells and pDC (Fig. 4A-C). To investigate the potential roles of APA in the the heterogeneity of immune cell populations in BC, a gene set enrichment analysis (GSEA) was performed. The results indicated that ERBB signaling pathway (normalized enrichment score, NES = -1.827, p.adj and FDR < 0.05), and JAK-STAT signaling pathway (NES = -2.054, p.adj and FDR < 0.05) were significantly down regulated in cluster 1 than that in cluster 2 (Figs. 4D and E, Supplementary Table 1), indicating that ERBB and JAK-STAT signaling pathways might play important roles in influencing the TIME of BC.

### Key APA regulatory factor CPSF3 is upregulated and an independent prognostic marker in BC

**Fig. 5:**
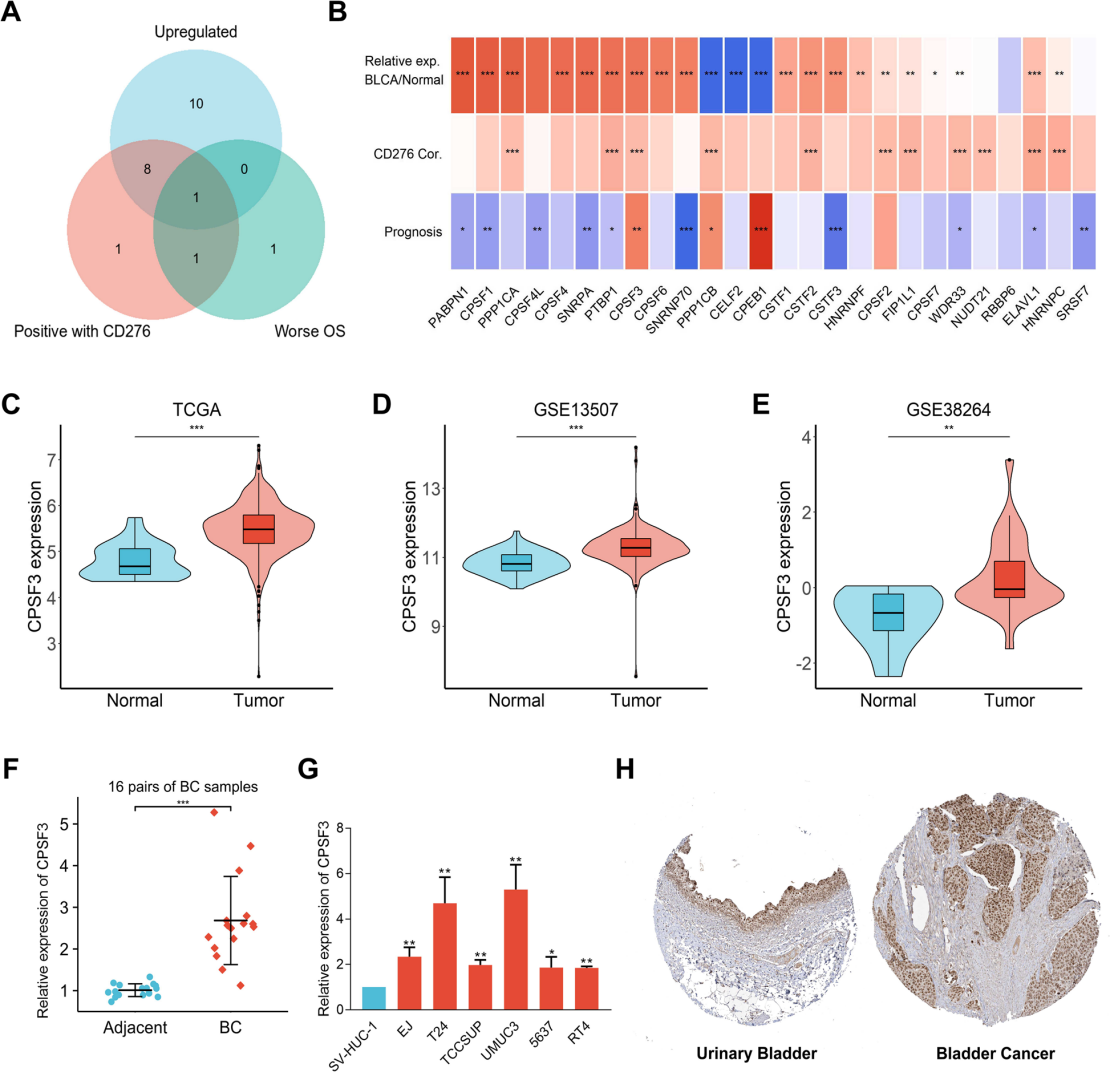
Analysis of the CPSF3 gene expression in BC. **A** The Venn diagram suggested that the upregulation of CPSF3 was an unfavorable prognostic factor and positively correlated with the CD276 expression in bladder cancer (BC). **B** The heat map visualized the relative expression of 26 APA regulators in BC/normal (row 1) and the correlations of 26 regulators with the CD276 expression (row 2) and prognosis (row 3). The red color indicates a high expression in BC, positively correlated with CD276 or unfavorable prognosis. *p < 0.05 and **p < 0.01. CPSF3 was upregulated in BC tissues compared to normal tissues.TCGA-BLCA cohort (**C**), GSE13507 (**D**), and GSE38264 (**E**). **F** Relative expression of CPSF3 was detected by qRT -PCR in 16 pairs of BC and normal tissues. **G** Compared with SV-HUC-1 cell line (normal), CPSF3 was highly expressed in EJ, T24, TCCSUP, UMUC3, 5637, RT4 cell lines (BC). **H** Immunohistochemistry staining indicated that, compared with normal bladder tissue (left), CPSF3 was significantly elevated in BC tissue (right) in the human protein atlas (antibody HPA034657, × 10). *p < 0.05, **p < 0.01

**Fig. 6:**
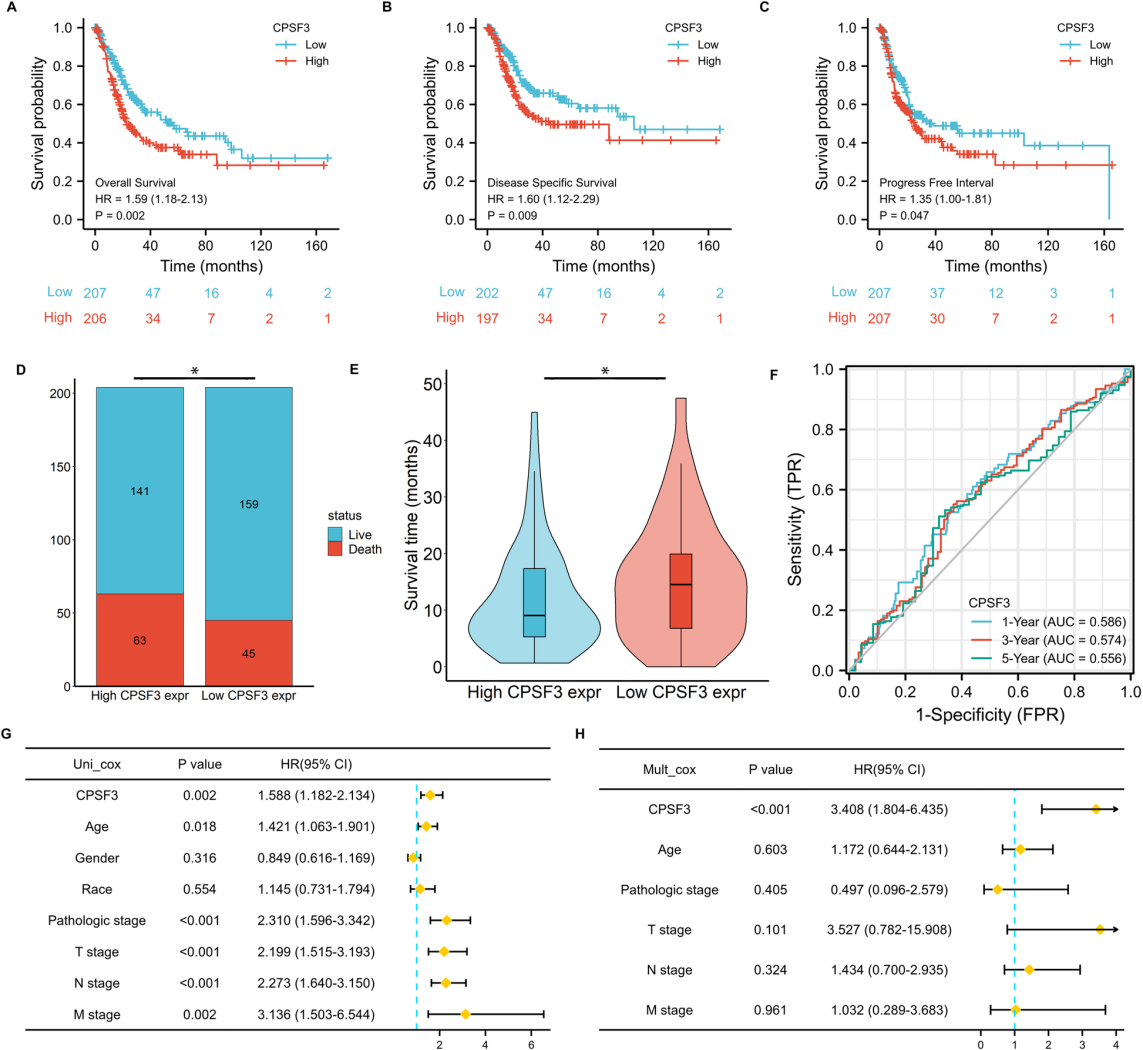
Upregulated CPSF3 expression is associated with poor outcomes of bladder cancer (BC). **A**, **B**, **C** The Kaplan–Meier analysis of BC patients with high and low CPSF3 expression level in the TCGA cohort for (**A**) overall surviva, (**B**) disease-specific surviva, (**C**) progress-free interval. **D**, **E** The stacked bar chart and violin plot visualized the proportion of live/death and survival time in high/low CPSF3 groups in BC patients. **F** Time-dependent receiver operating characteristics analysis of CPSF3. **G**, **H** Forest plots based upon the outcomes of univariate (**G**) and multivariate Cox regression (**H**) of CPSF3 expression and other clinicopathological factors. *p < 0.05

To further determine the key APA regulatory regulators in BC immune infiltration, an intersection of highly expressed APA regulators, poor prognostic significance, and positively correlated with CD276 expression among the 26 selected APA regulators was used (Figs. 5A). As shown in the Venn diagram, CPSF3 was identified as a key APA regulatory regulator (Figs. 5B). The correlation analysis showed that among the common immune checkpoint-related genes, CD276 and CPSF3 had the highest correlation coefficient (Supplementary Table 2).To further validate the expression of CPSF3 in BC, the expression profiles from TCGA and GEO database (GSE 13507 and GSE 38264) were analyzed (Fig. 5C-E). CPSF3 expression was significantly higher in tumor than that in the normal control group (TCGA, *P*< 0.001; GSE 13,507, *P* < 0.001; GSE38264, *P* < 0.01). The results of qRT-PCR and immunohistochemical assays showed that CPSF3 was higher in BC tissues and cell lines than that in adjacent non-tumor tissues and normal human ureteral epithelial cell lines (SV-HUC-1), respectively (Fig. 5F-H). These results indicated that CPSF3 is highly expressed in BC. We further assessed the prognostic value of CPSF3 in BC, the patients were divided into two groups using the median cut of CPSF3 level. The Kaplan–Meier survival analysis showed that higher CPSF3 was correlated with poor overall, disease-specific, and progress-free survival (Fig. 6A-C). Stacked bar graph and violin plot showed that the BC patients with higher CPSF3 expressions displayed a higher percentage of live/death and longer survival time (Fig. 6D and E). The operating characteristic curve (ROC) was used to evaluate the prognostic ability of CPSF3 expression in BC with 1-, 3-, 5- year survival (Fig. 6F). The univariate and multivariate analysis showed that CPSF3 is as an independent prognostic factor in BC (Fig. 6G and H).

### Correlation Analysis of CPSF3 With CD276 and Infiltrating Immune Cell

**Fig. 7:**
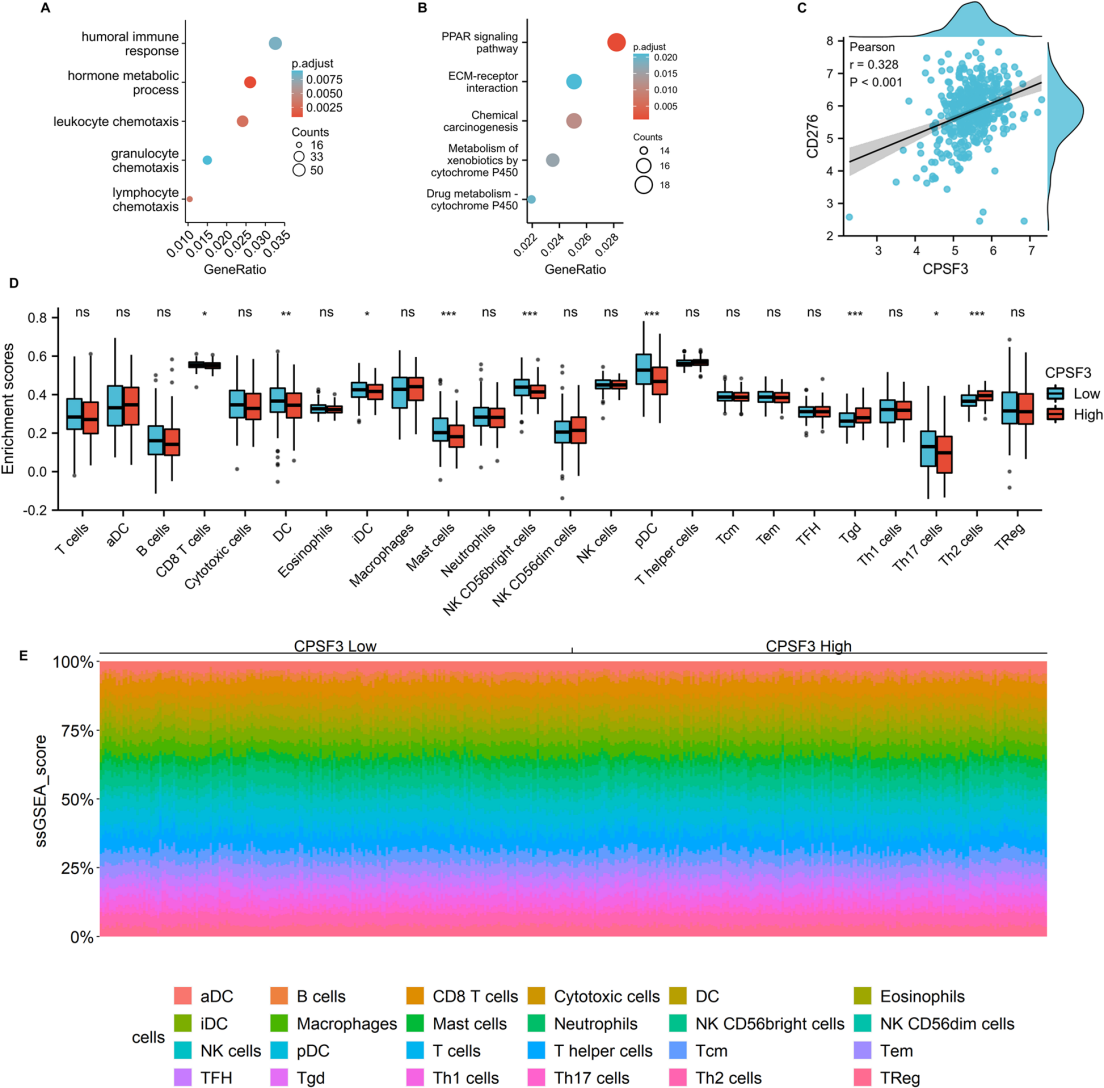
Correlation analysis of CPSF3 expression with CD276 and infiltrating immune cells in bladder cancer (BC). **A**, **B** The enriched Gene Ontology (**A**) and Kyoto Encyclopedia of Genes and Genomes signaling pathways (**B**) analysis of highly expressed CPSF3 in BC. **C** Pearson correlation analysis of CPSF3 expression and CD276 expression in BC. **D** The infiltrating levels of immune cells in high and low CPSF3 expression groups in BC patients. (**E**) The heat map visualized the percentage abundance of tumor-infiltrating immune cells in each sample. *p < 0.05, **p < 0.01, and ***p < 0.001

To further inverstigate the biological functions of CPSF3 in BC, we divided the patients into two groups using the median cut of CPSF3 level, and then performed GO and KEGG pathway enrichment analysis. The GO annotation identified major biological processes such as humoral immune response, hormone metabolic process, leukocyte chemotaxis, granulocyte chemotaxis, and lymphocyte chemotaxis (Fig. 7A, Supplementary Table 1). The KEGG pathway enrichment analysis revealed 33 pathways including PPAR signaling pathway, ECM-receptor interaction, chemical carcinogenesis, metabolism of xenobiotics by cytochrome P450, and drug metabolism-cytochrome P450 (Fig. 7B). The results indicated that CPSF3 may be involved in crucial signaling pathways in tumor immune microenvironment in BC. Correlation analysis indicated that the expression level of CPSF3 was positively correlated with CD276 expresion (Fig. 7C). The GSVA package was performed to calculate the proportion of immune cell subpopulations (Fig. 7D, E). As shown in the boxplot, the CPSF3 high expression group has higher proportions of Tgd and Th2 cells, while the low expression group has higher proportions of CD8 T cells, DC, iDC, Mast cells, NK CD56^bright^ cells, pDC, and Th17 cells.

## Discussion

BC is one of five tumor types (LUSC, LUAD, UCEC, BRCA, BC) that have the highest number of APA events [14]. The APA events in tumors result in 3′UTR shortening without any modification of the protein. Several APA associated genes have been identified as important regulators and prognostic indicators in multiple cancers [11, 12]. However, there are few studies evaluating the role of APA regulatory factors in the TIME in bladder cancer. In the present study, we analyzed the expression profile of 26 key APA regulatory factors in BC, and identified two distinctive subtypes of BC with different characteristics using consensus clustering method. Additionally, CPSF3 was identified as a potential key immune infiltration-related APA regulator in BC.

Recently studies revealed that APA events participated in modulating TIME of various tumors including breast cancer, gastric cancer, lower-grade glioma, lung adenocarcinoma et al. [15, 16]. Yet, the role of APA factors in BC TIME remains unclear. In our study, a comprehensive analysis of immune infiltration showed that cluster 1 exhibited higher infiltration of pDC and NK CD56^bright^ cells. In comparison, cluster 2 generally possessed higher proportions of both innate immune cells and adaptive immune cells. Unlike many other cancers types, our results suggest that BC subtype with generally lower immune cell infiltration has better overall and diseases specific survival and that APA regulatory factors may be important genetic signatures of BC.

In this study, we used consensus clustering analysis to characterize the role of APA regulators in TIME of BC, and found downregulated ERBB and JAK-STAT signaling in Cluster 1. Accumulating studies have revealed the oncogenic roles of the ERBB signaling in tumor progression in various types of cancer, including bladder, head and neck, breast, brain and gastrointestinal cancers [17, 18]. Aberrant expression of ERBB signaling proteins may result in an immunosuppressive microenvironment in tumors, enabling tumor cells to escape immune-mediated destruction [19]. Moreover, it has been found that aberrant activation of JAK signaling contributes to tumor invasion and metastasis [20]. Similarly to the ERBB signaling, the JSK-STAT signaling mediates multiple immune regulatory processes, which were involved in both tumor immune recognition and tumor immune evasion [21]. Together, our findings further reveal the potential role of APA regulators in regulating TIME in BC via the ERBB and JAK-STAT signaling pathways.

CD276 is a newly discovered immune checkpoint molecule that belongs to the B7 family. The protein B7-H3 encoded by CD276 has been verified to be highly expressed in various tumors including prostate cancer and non-small cell lung cancer [22]. Previous studies have shown that the expression of CD276 in tumor tissue is highly correlated with poor prognosis and survival time [23]. In this study, we collected 46 immune checkpoint-related molecules, including genes related to approved first-line immune checkpoint blockade drugs, of which CD276 and CPSF3 had the highest expression correlation. Next we verified the expression of CD276 in bladder cancer. CPSF3 encodes the 73-kD subunit of the CPSF complex and functions as an endonuclease that recognizes the pre-mRNA 3'-cleavage site AAUAAA prior to polyadenylation [24]. It has been implicated in multiple tumors including non-small cell lung cancer, colorectal cancer, AML and Ewing’s sarcoma et al. [25–27]. In this study, we first revealed that CPSF3 is correlated with unfavorable prognosis and the expression of CD276. Additionally, we found that BC patients with higher CPSF3 expression exhibit distinctive components of immune infiltrating cells compared that with lower CPSF3 expression. Recent studies have shown that APA is involved in the regulation of both innate and adaptive immune systems [28]. As a core APA regulator, CPSF3 may play important role in recognizing the polyadenylation signal sequence AAUAAA and cleaving the pre-mRNA, and gene expression in immune cells [29], implying that CPSF3 may regulates the function of immune cells in BC through alternative polyadenylation signals selection. These results suggest that CPSF3 is a potential target for BC immunotherapy.

There are several limitations in this study deserve to be mentioned. First, according to previous studies, 26 key APA regulators were included in this study, while there could be some other APA regulators be left out. Second, the clustering subtypes and ssGSEA analysis were calculated based on RNA-Seq data from the TCGA owing to the lack of sufficient data from our cohort. These results require further validation with single-cell sequencing or larger cohorts data. Third, additional in vitro and in vivo experiments to examine the underlying relationship between APA and TIME are needed.

In summary, this systemic study revealed the expression profile, prognostic value, and correlations with CD276 and TIME of APA regulators in BC. Moreover, we revealed a correleation between APA events and ERBB, and JAK-STAT signaling pathway, which might be implicated in the regulation of TIME in BC. Furthermore, we identified CPSF3 as a vital prognosis indicator for BC patients. These results may have potential implications for precise risk stratification of BC and the clinical trials on BC immunotherapy.

## Materials and methods

### Data acquisition

The gene expression profiles and corresponding clinical characteristics of the BC cohort were obtained from The Cancer Genomics Atlas database (TCGA; https://tcga-data.nci.nih.gov/tcga/). The dataset consisted of 408 patients diagnosed with BC and 19 normal control. Additional normal samples served as control were downloaded from the Genotype Tissue Expression dataset (GTEx; http://commonfund.nih.gov/GTEx/). Two microarray datasets GSE13507 and GSE38264 were downloaded from the Gene Expression Omnibus database (GEO; https://www.ncbi.nlm.nih.gov/geo/). The protein expression level of CPSF3 in BC compared to normal tissue was obtained from the Human Protein Atlas database (https://www.proteinatlas.org/). See Supplementary Table 3 for abbreviations of multiple tumor types, APA regulatory factors, and immune-related checkpoints.

### Bioinformatics analysis

Differential gene expression analyses were performed using the DESeq2 Bioconductor package (version 1.32.0) [30]. The Consensus Cluster Plus R package (version 4.1.0) was used for the clustering analysis (four-fifths of the total sample is drawn 100 times, and the maximum number of clusters is six) according to the expression level of selected APA regulators in BC patients [31]. Principal component analysis (PCA) and visualization were conducted by R package ggplot2 (version 3.3.5) [32]. GSEA analysis, GO analysis, KEGG analyses [33]were performed using the R package clusterProfiler (version 4.0.5) with hallmarks c2.cp.v7.2.symbols from MSigDB Collections. Obtained p-values for the permutation tests were adjusted for multiple testing using the Benjamini–Hochberg correction [34]. A pathway was considered significantly enriched with FDR *p* value < 0.05. Immune infiltration of BC was estimated using the ssGSEA method of GSVA package (version 1.34.0) [35]. We divided TCGA BLCA patients into two pairs of comparison groups based on the median cut of CPSF3 level and clustering results, respectively. Cell subtypes with *p* < 0.05 were considered key immune cells. The results visualization of relative fractions was done using the R packages ggplot2 (version 3.3.5) and ComplexHeatmap (version 2.8.0) [36]. Survival analyses were performed using R packages survival (version 3.2–13) and survminer (version 0.4.9) [37, 38]. Cox proportional hazards regression models were adopted to identify the prognostic value of CPSF3 in BC.

### Clinical samples, cell lines, and qRT -PCR analysis

Sixteen BC tissue specimens and paired adjacent non-tumorous tissue were obtained from patients subjected to radical resection of bladder cancer between April and December 2021 at Huazhong University of Science and Technology affiliated Union Hospital. All of the patients have been pathologically and clinically diagnosed. This research was approved by the Ethics Committee of Huazhong University of Science and Technology affiliated Union Hospital. All written informed consent was obtained as well. To select adjacent non-tumor bladder tissues, grossly normal mucosa from the farthest resection margin was carefully excised and subjected to frozen section evaluation in order to exclude dysplasia and the presence of carcinoma cells. The surface squamous epithelium of an adjacent area was then carefully peeled off from the submucosa and placed immediately in liquid nitrogen. BC cell lines including SV-HUC-1, EJ, T24, TCCSUP, UMUC3, 5637, and RT4 were obtained from the Cell Bank of the Chinese Academy of Sciences. Total RNA was extracted using the RNA isolater Reagent (Vazyme) and reverse transcribed using the HiScript cDNA Synthesis Kit (Vazyme). Quantitative real-time PCR (qRT-PCR) was performed on StepOne Plus real-time PCR system (Life Technologies) with ChamQ SYBR qPCR Master Mix (Vazyme). GAPDH served as the housekeeping gene. Primers and clinical information are listed in Supplementary Tables 4, 5.

### Statistical analysis

All statistics were carried out with R software (version 4.1.0). Normality was evaluated using the Shapiro–Wilk normality test. Mann–Whitney U Test was performed if expression data from TCGA cohort failed test for normality. CPSF3 expression level in 16 pairs of BC and adjacent control tissues was calculated by dependent t-test for paired samples. Group comparisons of two or more groups were performed by Wilcoxon rank sum test and Kruskal–Wallis test, respectively. Expression correlations between APA regulatory factors with immune checkpoints-related genes were statistically evaluated using Pearson correlation analysis. Survival analysis was performed using the Logrank test. Univariate and multivariate analyses were conducted using Cox regression models to identify independent prognostic predictors for this cohort. Statistical significance were indicated as * *P* < 0.05, ** *P* < 0.01, and *** *P* < 0.001.

## Supplementary Information

**Additional file 1: Supplementary Figure 1.** Consensus clustering for APA regulators in bladder cancer (BC). (A–D) Four heat maps exhibit the clustering matrix for APA regulators in BC patients for k = 3, 4, 5, and 6. The tighter and clearer the clusters are, the more optimal the cluster. (E) Delta area curve of consensus clustering for k = 2–6. (F) Principal component analysis of BC patients' APA regulator expression profiles demonstrates two patient clusters.

**Additional file 2: Supplementary Table 1.** The results of the GSEA.

**Additional file 3: Supplementary Table 2.** Correlation statistics between CPSF3 and immune checkpoint-related genes.

**Additional file 4: Supplementary Table 3.** The abbreviations of cancer types, alternative polyadenylation regulators and immune-related checkpoints in this study.

**Additional file 5: Supplementary Table 4.** The patients’ clinical information (*n* = 16) in this study.

**Additional file 6: Supplementary Table 5.** The oligonucleotides used in this study.∗F, forward primer; R, reverse primer.

## Data Availability

The datasets analysed during the current study are available in the The Cancer Genomics Atlas database [TCGA; https://tcga-data.nci.nih.gov/tcga/], the Genotype Tissue Expression dataset [GTEx; http://commonfund.nih.gov/GTEx/], the Gene Expression Omnibus database [GEO; https://www.ncbi.nlm.nih.gov/geo/], The Human Protein Atlas[https://www.proteinatlas.org/]. The names of the repository/repositories and accession number(s) can be found in the article/Supplementary Material.
