# Supplementary figures and images for: Comprehensive analysis of alternative polyadenylation regulators concerning CD276 and immune infiltration in bladder cancer
Source: BMC Cancer. 2022 Sep 29;22:1026. doi: 10.1186/s12885-022-10103-7 (PMC9520876; doi:10.1186/s12885-022-10103-7)

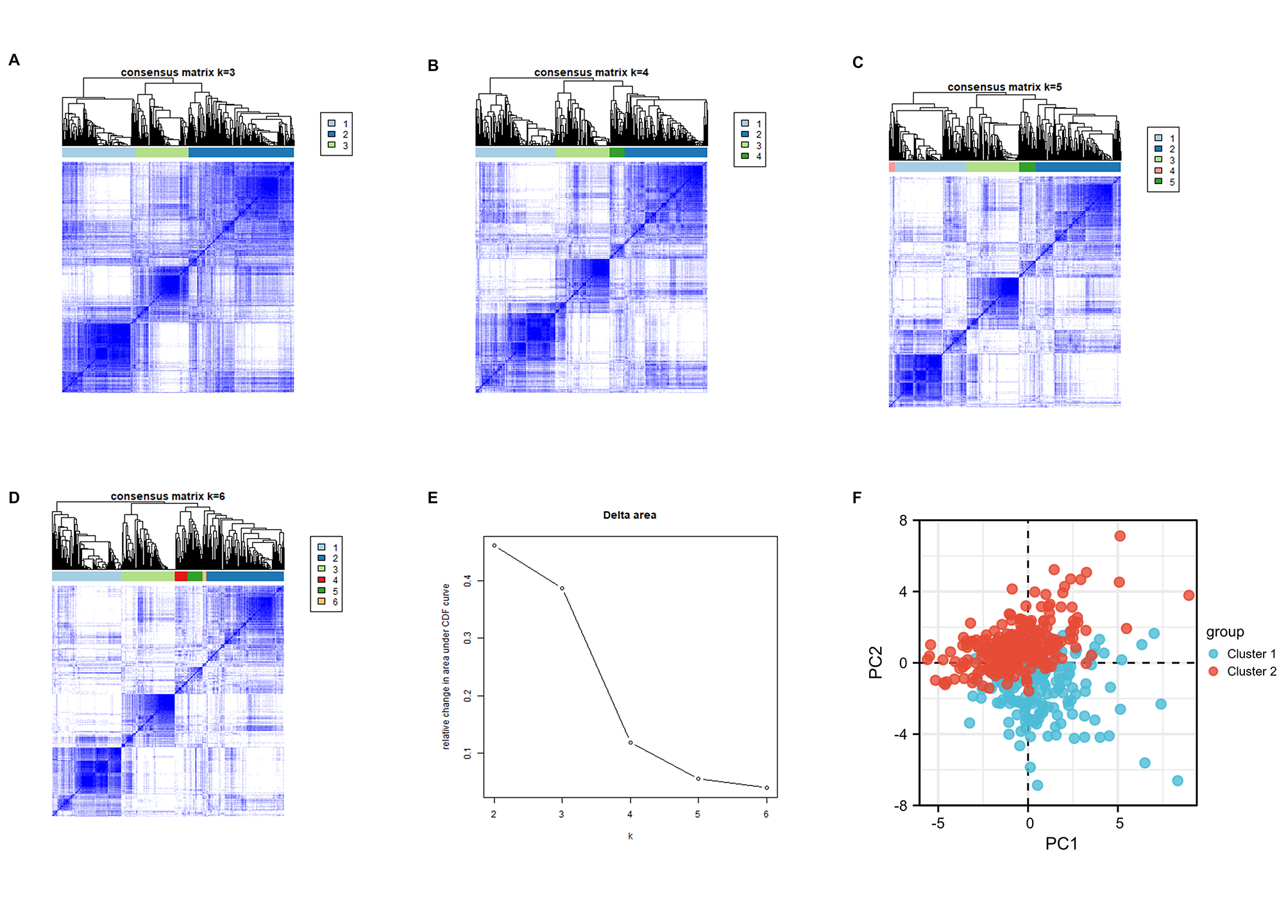

Supplement: Supplementary file 1 — Additional file 1: Supplementary Figure 1. Consensus clustering for APA regulators in bladder cancer (BC). (A–D) Four heat maps exhibit the clustering matrix for APA regulators in BC patients for k = 3, 4, 5, and 6. The tighter and clearer the clusters are, the more optimal the cluster. (E) Delta area curve of consensus clustering for k = 2–6. (F) Principal component analysis of BC patients' APA regulator expression profiles demonstrates two patient clusters. [file 12885_2022_10103_MOESM1_ESM.tif]
