# Supplementary material for: Comprehensive analysis of alternative polyadenylation regulators concerning CD276 and immune infiltration in bladder cancer
Source: BMC Cancer. 2022 Sep 29;22:1026. doi: 10.1186/s12885-022-10103-7 (PMC9520876; doi:10.1186/s12885-022-10103-7)
Supplement: Supplementary file 4 — Additional file 4: Supplementary Table 3. The abbreviations of cancer types, alternative polyadenylation regulators and immune-related checkpoints in this study. [file 12885_2022_10103_MOESM4_ESM.docx]

| Supplementary Table 3. The abbreviations of multiple cancer types, alternative polyadenylation regulators, and immune-related checkpoints in this study. | |
| --- | --- |
| Abbreviation  (cancer types) | Full name |
| BLCA | Bladder Urothelial Carcinoma |
| BRCA | Breast invasive carcinoma |
| LUAD | Lung adenocarcinoma |
| LUSC | Lung squamous cell carcinoma |
| UCEC | Uterine Corpus Endometrial Carcinoma |
| Abbreviation  (alternative polyadenylation regulators) | Full name |
| PABPN1 | poly(A) binding protein nuclear 1 |
| CPSF1 | cleavage and polyadenylation specific factor 1 |
| PPP1CA | protein phosphatase 1 catalytic subunit alpha |
| CPSF4L | cleavage and polyadenylation specific factor 4 like |
| CPSF4 | cleavage and polyadenylation specific factor 4 |
| SNRPA | small nuclear ribonucleoprotein polypeptide A |
| PTBP1 | polypyrimidine tract binding protein 1 |
| CPSF3 | cleavage and polyadenylation specific factor 3 |
| CPSF6 | cleavage and polyadenylation specific factor 6 |
| SNRNP70 | small nuclear ribonucleoprotein U1 subunit 70 |
| PPP1CB | protein phosphatase 1 catalytic subunit beta |
| CELF2 | CUGBP Elav-like family member 2 |
| CPEB1 | cytoplasmic polyadenylation element binding protein 1 |
| CSTF1 | cleavage stimulation factor subunit 1 |
| CSTF2 | cleavage stimulation factor subunit 2 |
| CSTF3 | cleavage stimulation factor subunit 3 |
| HNRNPF | heterogeneous nuclear ribonucleoprotein F |
| CPSF2 | cleavage and polyadenylation specific factor 2 |
| FIP1L1 | factor interacting with PAPOLA and CPSF1 |
| CPSF7 | cleavage and polyadenylation specific factor 7 |
| WDR33 | WD repeat domain 33 |
| NUDT21 | nudix hydrolase 21 |
| RBBP6 | RB binding protein 6, ubiquitin ligase |
| ELAVL1 | ELAV like RNA binding protein 1 |
| HNRNPC | heterogeneous nuclear ribonucleoprotein C |
| SRSF7 | serine and arginine rich splicing factor 7 |
| Abbreviation  (immune-related checkpoints) | Full name |
| CD276 | CD276 molecule |
| CD274/PD-L1 | CD274 molecule/Programmed cell death 1 ligand 1 |
| CD24 | CD24 molecule |
| CD47 | CD47 molecule |
| CTLA4 | cytotoxic T-lymphocyte associated protein 4 |
| HAVCR2 | hepatitis A virus cellular receptor 2 |
| LAG3 | lymphocyte activating 3 |
| PDCD1 | programmed cell death 1 |
| SIGLEC15 | sialic acid binding Ig like lectin 15 |
| TIGIT | T cell immunoreceptor with Ig and ITIM domains |
| BTLA | B and T lymphocyte associated |
| BTN2A1 | Butyrophilin subfamily 2 member A1 |
| CD160 | CD160 molecule |
| CD209 | CD209 molecule |
| CD226 | CD226 molecule |
| CD28 | CD28 molecule |
| CD40 | CD40 molecule |
| CD40LG | CD40 ligand |
| CD86 | CD86 molecule |
| CD96 | CD96 molecule |
| CEACAM1 | CEA cell adhesion molecule 1 |
| HLA-A | Major histocompatibility complex, class I, A |
| HLA-B | Major histocompatibility complex, class I, B |
| HLA-C | Major histocompatibility complex, class I, C |
| HLA-DMB | Major histocompatibility complex, class II, DM beta |
| HLA-DPB1 | Major histocompatibility complex, class II, DP beta 1 |
| HLA-DQA1 | Major histocompatibility complex, class II, DQ alpha 1 |
| HLA-DQB1 | Major histocompatibility complex, class II, DQ beta 1 |
| HLA-DRA | Major histocompatibility complex, class II, DR alpha |
| HLA-F | Major histocompatibility complex, class I, F |
| ICOS | Inducible T cell costimulator |
| ICOSLG | Inducible T cell costimulator ligand |
| IDO1 | Indoleamine 2,3-dioxygenase 1 |
| KIR2DL1 | Killer cell immunoglobulin like receptor, two Ig domains and long cytoplasmic tail 1 |
| KIR2DL3 | Killer cell immunoglobulin like receptor, two Ig domains and long cytoplasmic tail 2 |
| KIR3DL2 | Killer cell immunoglobulin like receptor, two Ig domains and long cytoplasmic tail 3 |
| LGALS9 | Galectin 9 |
| PDCD1LG2 | Programmed cell death 1 ligand 2 |
| PVR | PVR cell adhesion molecule |
| SIRPA | Signal regulatory protein alpha |
| TDO2 | Tryptophan 2,3-dioxygenase |
| TNFRSF14 | Tumor necrosis factor receptor superfamily member 14 |
| TNFRSF18 | Tumor necrosis factor receptor superfamily member 18 |
| TNFSF14 | Tumor necrosis factor superfamily member 14 |
| TNFSF18 | Tumor necrosis factor superfamily member 18 |
| TNFSF9 | Tumor necrosis factor superfamily member 9 |
