# Supplementary material for: Comprehensive analysis of alternative polyadenylation regulators concerning CD276 and immune infiltration in bladder cancer
Source: BMC Cancer. 2022 Sep 29;22:1026. doi: 10.1186/s12885-022-10103-7 (PMC9520876; doi:10.1186/s12885-022-10103-7)
Supplement: Supplementary file 5 — Additional file 5: Supplementary Table 4. The patients’ clinical information (n = 16) in this study. [file 12885_2022_10103_MOESM5_ESM.docx]

| Supplementary Table 4. The patients’ clinical information （n=16）in this study. | | | | |
| --- | --- | --- | --- | --- |
| No. | Age | Sex | Pathological diagnosis | Clinical TNM Stage |
| 1 | 62 | male | bladder urothelial carcinoma | T2N0M0 |
| 2 | 69 | male | bladder urothelial carcinoma | T4NxM0 |
| 3 | 74 | male | bladder urothelial carcinoma | T3N1M0 |
| 4 | 59 | male | bladder urothelial carcinoma | T2N0M0 |
| 5 | 78 | male | bladder urothelial carcinoma | T2N0M0 |
| 6 | 64 | male | bladder urothelial carcinoma | T3N1M0 |
| 7 | 60 | male | bladder urothelial carcinoma | T2NxM0 |
| 8 | 65 | male | bladder urothelial carcinoma | T2N0M0 |
| 9 | 71 | male | bladder urothelial carcinoma | T3N2Mx |
| 10 | 58 | male | bladder urothelial carcinoma | T2N0M0 |
| 11 | 63 | male | bladder urothelial carcinoma | T3N2M0 |
| 12 | 81 | male | bladder urothelial carcinoma | T2N0M0 |
| 13 | 76 | male | bladder urothelial carcinoma | T3N1M0 |
| 14 | 72 | male | bladder urothelial carcinoma | T3NxM0 |
| 15 | 67 | male | bladder urothelial carcinoma | T2N0M0 |
| 16 | 63 | male | bladder urothelial carcinoma | T2N0M0 |
