# Supplementary material for: Comprehensive analysis of alternative polyadenylation regulators concerning CD276 and immune infiltration in bladder cancer
Source: BMC Cancer. 2022 Sep 29;22:1026. doi: 10.1186/s12885-022-10103-7 (PMC9520876; doi:10.1186/s12885-022-10103-7)
Supplement: Supplementary file 6 — Additional file 6: Supplementary Table 5. The oligonucleotides used in this study.∗F, forward primer; R, reverse primer. [file 12885_2022_10103_MOESM6_ESM.docx]

| Supplementary Table 5. The oligonucleotides used in this study. | | |
| --- | --- | --- |
| Name |  | Sequence (5’ > 3’) |
| GAPDH | Forward priner | GGAGCGAGATCCCTCCAAAAT |
|  | Reverse primer | GGCTGTTGTCATACTTCTCATGG |
| CPSF3 | Forward priner | ATGTCTGCGATTCCTGCTGAG |
|  | Reverse primer | ATCCCACAGTCGAGCATTATTTT |
